# Diagnosing pubovisceral avulsions: a systematic review of the clinical relevance of a prevalent anatomical defect

**DOI:** 10.1007/s00192-012-1805-0

**Published:** 2012-05-12

**Authors:** Karin Lammers, Jurgen J. Fütterer, Mathias Prokop, Mark E. Vierhout, Kirsten B. Kluivers

**Affiliations:** 1 Department of Obstetrics and Gynaecology (791), Radboud University Nijmegen Medical Centre, P.O. Box 9101, 6500 HB Nijmegen, The Netherlands; 2 Department of Radiology (667), Radboud University Nijmegen Medical Centre, Nijmegen, The Netherlands

**Keywords:** Levator ani, Levator defect, MR imaging, Perineal ultrasonography, Pubovisceral avulsion

## Abstract

The aims of this systematic literature review were to assess whether the detection of pubovisceral avulsions using magnetic resonance (MR) imaging or perineal ultrasonography was clinically relevant in women with pelvic floor dysfunction and to evaluate the relation with anatomy, symptoms, and recurrence after surgery. We performed a systematic literature review using three bibliographical databases (PubMed, Embase, and CINAHL) as data sources. Clinical studies were included in which pubovisceral avulsions were studied in relation to pelvic organ prolapse (POP) stage, pelvic floor symptoms, and/or recurrence of POP after surgery. Ultimately, 21 studies met the inclusion criteria. POP stage and recurrence of POP after surgery were strongly associated with pubovisceral avulsions. Contradictory results were found regarding the relation between pubovisceral avulsions and urinary symptoms and symptoms of anorectal dysfunction. Pubovisceral avulsions, as diagnosed by MR imaging or perineal ultrasonography, are associated with higher stages of POP and recurrence of POP after surgery.

## Introduction

Pelvic floor dysfunction is an often used term that covers many different conditions including pelvic organ prolapse (POP), urinary incontinence (UI), and anorectal dysfunction. POP has a significant negative impact on women’s quality of life, affects physical well-being, psychological and sexual functioning, and causes occupational and social restraints [1]. Up to 20 % of the general female population will have symptoms of POP and/or UI significant enough to require surgery [2–5]. Both conditions often occur concurrently: up to 40 % of POP patients have preoperative concomitant UI [1, 6]. Of the women undergoing POP surgery, almost one third will develop postoperative recurrence for which additional surgery is needed. The highest recurrence rates are reported in the anterior vaginal compartment [2, 7]. Important risk factors for the development of POP are a family history of POP and vaginal delivery [8], and over the past decade levator ani muscle injury emerged as a major contributing factor in POP pathophysiology. This type of injury is only observed in women who have given birth vaginally or have at least entered the second stage of labor. Risk factors for levator ani muscle injury are forceps delivery, length of second stage of labor, and large fetal head circumference [9–12]. Besides being associated with an increased risk of POP, levator ani muscle injury has been reported to lead to an increased risk of recurrence after POP surgery [13, 14].

The prevalence of levator ani muscle injury is reported to be up to 36 % in vaginally parous women and presents as a detachment, i.e., avulsion, of the pubovisceral component of the levator ani muscle from the pubis [15–17]. These pubovisceral avulsions can be observed as a complete loss of connection to the pubis or as a partial detachment with apparent loss of muscle bulk, both either unilateral or bilateral. Pubovisceral avulsions can be visualized using magnetic resonance (MR) imaging [13] or three-dimensional (3-D) perineal ultrasonography [18]. MR imaging is a noninvasive diagnostic tool that allows for detailed evaluation of all soft tissue structures of the pelvic support system. Perineal ultrasonography is increasingly used in urogynecology and provides four-dimensional (4-D, i.e., 3-D +temporal information) assessment of the pelvic floor during routine clinical practice. Both MR imaging and perineal ultrasonography require post-processing of the obtained image data, for which significant training is needed. Which of these diagnostic methods is best for diagnosing pubovisceral avulsions is still under investigation.

Various studies have been conducted with regard to pubovisceral avulsions and the associated risks and specific symptoms. The aims of this systematic literature review were to assess whether the detection of pubovisceral avulsions by MR imaging or perineal ultrasonography was clinically relevant in women with pelvic floor dysfunction and to evaluate if there was a relation with anatomy and symptoms.

## Materials and methods

A systematic literature search was performed by a clinical researcher (KL) and a senior librarian. The electronic databases PubMed, Embase, and CINAHL were searched from inception up to 27 September 2011. The search and selection of the literature were restricted to publications written in Western languages. To capture all relevant articles on the clinical relevance of pubovisceral avulsions, as diagnosed by MR imaging or perineal ultrasonography, we chose the following strategy: search term combinations were adapted for each database and consisted of Medical Subject Headings (MeSH), thesaurus terms and CINAHL headings, text words and word variations for the terms “pelvic floor,” “MR imaging,” “ultrasonography,” and “physical examination.” The entire strings of search terms are depicted in Appendices 1, 2, and 3. Due to the large variability of terms that indicate pubovisceral avulsions, e.g., detachment, disconnection, tearing off, and severing, we did not attempt to include the corresponding terms in the search strategy but used this as an inclusion criterion. Hereby, the initial search was as sensitive as possible.

Articles identified by the literature search were included in our systematic review in case they reported on pubovisceral avulsions diagnosed by at least one of the two diagnostic methods: MR imaging and perineal ultrasonography. Articles were included if they concerned clinical studies that provided data on POP status, pelvic floor symptoms, or recurrence of POP after surgery. Pelvic floor symptoms had to be documented using standardized questions or validated (quality of life) questionnaires. Recurrence of POP after surgery and POP status had to be documented with a standardized method, such as the Pelvic Organ Prolapse Quantification (POP-Q) [19] or Baden-Walker system, or stated as number of reoperations. Letters, commentaries, and editorial notes were excluded.

All studies were evaluated by title and abstract according to the inclusion and exclusion criteria by KL. If necessary, full text articles were evaluated. After this preselection, a final decision on inclusion or exclusion was made in consensus with an experienced pelvic floor specialist (KK). Reference lists of relevant retrieved studies were cross-checked to identify additional studies that had been overlooked in the database search.

The full text articles were evaluated to collect data on study design, aim of the study, sample size, study population, control group, parity, age, diagnostic method(s), POP staging, previous prolapse surgeries, number of reoperations, and the method(s) of pelvic floor symptom assessment.

To report pubovisceral avulsions, scoring systems have previously been developed by other research groups. In MR imaging, defect severity is scored in both muscle sides separately, ranging from 0 (no defect) to 3 (complete muscle loss). A summed score for the two sides (0–6) can then be assigned and grouped as no defect (0), minor defect (1–3), or major defect (4–6, or a unilateral score of 3) [20]. For perineal ultrasonography, the integrity of the pubovisceral muscle is evaluated in the axial plane using multislice imaging, i.e., tomographic ultrasound imaging (TUI). A set of eight tomographic slices are evaluated at intervals of 2.5 mm, in which both muscle sides are scored separately, resulting in a defect score ranging from 0 (no defect) to 16 (complete bilateral avulsion). A complete avulsion is diagnosed if the reference slice, i.e., the slice that represents the plane of minimum hiatal dimensions, as well as the two slices immediately cranial to this plane show an avulsion. Partial avulsion is diagnosed when any of the slices are abnormal, without the patient being classified as having a complete avulsion [21, 22].

## Results

**Fig. 1:**
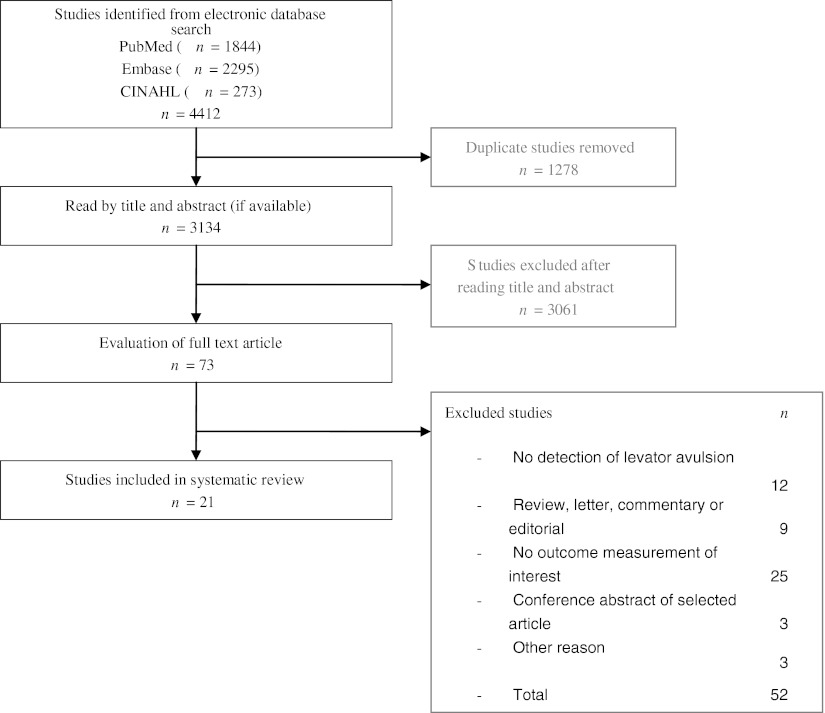
Selection process of studies included in the systematic review. *n* number of articles

The PubMed search revealed 1,844 articles. The Embase and CINAHL searches found an additional 1,171 and 119 articles, respectively. In total, 3,134 articles were checked for eligibility. No additional studies were identified by cross-checking reference lists. A flowchart of the selection procedure is presented in Fig. 1. Eight studies reported on detection of pubovisceral avulsion using MR imaging [13, 15, 23–28]. Twelve studies used perineal ultrasonography as the diagnostic method [14, 17, 22, 29–37]. Apart from these 20 studies that used one diagnostic method for the evaluation of pubovisceral avulsions, we identified 4 studies that compared the use of different diagnostic methods. Of these, two studies compared perineal ultrasonography with palpation of the pubovisceral muscle defect [38, 39]. In both studies, the detection of pubovisceral avulsions was the outcome measure of interest. One study compared MR imaging with palpation [40] and one study compared ultrasonography with MR imaging [41]. In these studies, the objective was to determine the agreement in the detection of abnormalities in the pubovisceral muscle. Of the latter four studies that evaluated two diagnostic methods, only one compared their results with our outcomes of interest [38] and was therefore the only article included in this review that used two different diagnostic methods. Ultimately, 21 articles could be included in our systematic literature review. Study designs, group characteristics, diagnostic methods, and outcome measures are summarized in Table 1 [13–15, 17, 22–38]. Apart from two studies published in 2003 [15, 23], all other articles have been published after 2005.

**Table 1:** Characteristics of clinical studies reporting on diagnosing pubovisceral avulsions

First author and year of publication	*n* in study	Study population	Age, years^a^	Parity, number^a^	Study design	Diagnostic method	Outcome measure
Abdool 2009 [29]	361	Urogynecological complaints	55 [19–89]	88 % vaginally parous	RC	US	POP anatomy and symptoms
Adekanmi 2003 [23]	70	Cases: symptomatic POP ≥ stage II	62	2 [1–5]	CC	MR	POP anatomy and symptoms
		Controls: nulliparae		Nulliparous			
Chantarasorn 2011 [30]	393	Urogynecological complaints	54 [18–89]	2 [0–9]	RC	US	Symptoms of anorectal dysfunction
DeLancey							
2003 [15]	240	Cases: primiparae with SUI (*n* = 80)	30.0 (5.7)	Primiparous	CC	MR	Urinary symptoms
		Control groups:					
		Continent primiparae (*n* = 80)	29.8 (4.4)	Primiparous			
		Nulliparae (*n* = 80)	29.2 (5.5)	Nulliparous			
2007 [13]	286	Cases: POP ≥ stage II (*n* = 151)	56.4 (12.9)	3.0 (1.8)	CC	MR	POP anatomy and symptoms
		Controls: POP < stage II (*n* = 135)	56.6 (13.0)	2.3 (1.5)			
Dietz							
2006 [17]	333	Urogynecological complaints	52.8 (13.3)	3 [0–8]	PC	US	Urinary symptoms, POP anatomy and symptoms
2007 [31]	262	Urogynecological complaints	54 [26–82]	2.7 [1–6]	RC	US	Urinary symptoms, POP anatomy and symptoms
2008 [14]	781	Urogynecological complaints	53 [15–89]	2 [0–12]	RC	US	POP anatomy and symptoms
2008 [38]	107	Urogynecological complaints	55.5 [17–85]	2 [0–8]	PC	US, palpation	POP anatomy and symptoms
2009 [33]	420	Urogynecological complaints	55 [17–87]	3 [0–10]	RC	US	Urinary symptoms, POP anatomy and symptoms
2010 [22]	753	Urogynecological complaints	55 [18–89]	2 [0–10]	RC	US	POP anatomy and symptoms
2010 [32]	83	Status after anterior colporrhaphy	61 [31–86]	3 [1–9]	PC	US	Recurrence of POP
Heilbrun 2010 [24]	206	Primiparae	27.8 (6.1)	Primiparous	CC	MR	Urinary symptoms, symptoms of anorectal dysfunction, POP anatomy and symptoms
Lewicky-Gaupp 2010 [25]	26	Cases: older women with FI (*n* = 8)	71.6 [63–85]	2.6 (1.2)	CC	MR	Symptoms of anorectal dysfunction
		Control groups:					
		Older continent (*n* = 9)	71.6 [60–88]	2.8 (1.7)			
		Younger continent (*n* = 9)	28.7 [20–41]	0.2 (0.4)			
Model 2010 [34]	737	Urogynecological complaints	54.9 [17–89]	2 [0–9]	RC	US	Recurrence of POP
Morgan							
2007 [27]	286	Cases: POP ≥ stage II (*n* = 151)	56.4 (12.9)	3.0 (1.8)	CC	MR	Symptoms of anorectal dysfunction
		Controls: POP ≤ stage I (*n* = 135)	56.6 (13.0)	2.3 (1.5)			
2010 [26]	151	POP ≥ stage II	56.4 (12.9)	3.0 (1.8)	CC	MR	Urinary symptoms
2011 [28]	83	Primary surgery for POP ≥ stage II	58.5 (11.2)	82.6 % of cases ≥ 2 children	CC	MR	POP anatomy and symptoms, recurrence of POP
Rodrigo 2011 [36]	967	Urogynecological complaints	56 [17–90]	90 % vaginally parous	RC	US	Symptoms of anorectal dysfunction
Weemhoff 2011 [35]	152	Status after anterior colporrhaphy	61.4 (10.1)	2.3 (1.1)	PC	US	Recurrence of POP
Wong^b^ 2011 [37]	220	Status after anterior vaginal mesh	64.4 [32–89]	3 [0–10]	RC	US	Recurrence of POP

*n* number of patients, *SD* standard deviation, *RC* retrospective cohort, *CC* case-control, *PC* prospective cohort, *US* ultrasound, *MR* magnetic resonance imaging, *POP* pelvic organ prolapse, *SUI* stress urinary incontinence, *FI* fecal incontinence

^a^Mean (SD) or median [range]

^b^Conference abstract, (so far) no full text article available

Tables 2, 3, 4 and 5 provide an overview of the studies assessing pubovisceral avulsions in relation to urinary symptoms, symptoms of anorectal dysfunction, POP anatomy and symptoms, and recurrence of POP after surgery, respectively.

**Table 2:** Urinary symptoms in women assessed for pubovisceral avulsions

First author	*n* with levator avulsion		Percentage of women of study population with levator avulsion	Conclusion
DeLancey [15]	Cases:	23	29 %	Primiparous women with SUI were twice as likely to have a muscle abnormality than primiparae without SUI. No avulsions were identified in nulliparous women
	Control groups:			
	Non-SUI primiparae	9	11 %	
	Nulliparae	0	0 %	
Dietz [17]	Complete	46	14 %	No association was found between complete avulsion and urodynamic findings or symptoms of bladder dysfunction except for frequency (*p* = 0.02)
Dietz [31]	Complete	50	19 %	Sixty-two percent of women with complete avulsions complained of SUI. Defect score was significantly higher in women with symptoms of urinary frequency (*p* = 0.05)
Dietz [33]	Complete	104	25 %	Women with complete avulsions were less likely to suffer from SUI (*p* < 0.001) and USI (*p* = 0.065), but more likely to show signs of voiding dysfunction (*p* = 0.005)
Heilbrun [24]	Major	20	10 %	There was no relation between major avulsion and (S)UI based on MESA compared to women with minor or no avulsion
Morgan [26]	Major	83	55 %	SUI symptoms were least present in women with major avulsions and most frequently reported by women with minor avulsions
	Minor	24	16 %	

*n* number of patients, *(S)UI* (stress) urinary incontinence, *p value* *p* value for difference between avulsion and outcome measure assessed, *USI* urodynamic stress incontinence, *MESA* medical, epidemiological, and social aspects of aging

Studies on the relation between urinary symptoms and pubovisceral avulsions yielded contradictory results (Table 2). DeLancey et al. [15] found that the prevalence of pubovisceral avulsions was more than twice as high in primiparous women with stress urinary incontinence (SUI) than in primiparous women without SUI. Dietz et al. reported that women with urogynecological complaints and pubovisceral avulsions more often suffered from urinary frequency [17] and voiding dysfunction [33]. They also found that women over the age of 50 were less likely to suffer from SUI [33]. Morgan et al. [26] studied women with POP ≥ stage II and found that women with major defects had the lowest risk of SUI symptoms, while this risk was the highest in women with minor defects. Heilbrun et al. [24] reported no association between complaints of (S)UI and levator ani defect status.

Two of the available studies on anorectal dysfunction found that women with major or complete pubovisceral avulsions were more likely to report symptoms of anorectal dysfunction than women with minor or no avulsions (Table 3). This association was found in women who had suffered an anal sphincter tear during delivery [24] and “older” women [25]. For women with POP ≥ stage II and for women with urogynecological complaints, i.e., women with symptoms of lower urinary tract and pelvic floor dysfunction, no significant relation between symptoms of anorectal dysfunction and pubovisceral avulsions could be found [27, 30]. Rodrigo et al. [36] reported that the prevalence of rectal intussusception (RI) was higher among women with pubovisceral avulsions.

**Table 3:** Symptoms of anorectal dysfunction and the relation with pubovisceral avulsions

First author	*n* with levator avulsion		Percentage of women of study population with levator avulsion	Conclusion
Chantarasorn [30]	Complete bilateral	38	10 %	Both unilateral and bilateral complete avulsions were not associated with FI, fecal urgency, or flatus incontinence
	Complete unilateral	39	10 %	
Heilbrun [24]	Major	20	10 %	Women with major avulsions had a higher prevalence of FI based on FISI questionnaire (*p* = 0.006) compared to women with no or minor avulsions
Lewicky-Gaupp [25]	OI	6	75 %	Older women with FI were more likely to have avulsions than women without FI
	OC	2	22 %	
	YC	1	11 %	
	Overall	9	35 %	
Morgan [27]	Cases:			There was no relation between levator ani defect status and symptom severity of anal incontinence and difficult defecation among women with POP
	Major	83	55 %	
	Minor	24	16 %	
	Controls:			
	Major	21	16 %	
	Minor	30	22 %	
Rodrigo [36]	Complete	209	22 %	Levator ani avulsion was more common among women with RI (*p* = 0.003). On multivariable analysis, there was no independent effect of avulsion

*n* number of patients, *OI* older incontinent, *OC* older continent, *YC* young continent, *FI* fecal incontinence, *FISI* Fecal Incontinence Severity Index, *p value* *p* value for difference between avulsion and outcome measure assessed, *POP* pelvic organ prolapse, *RI* rectal intussusception

Eleven studies reported on POP anatomy and symptoms in relation to pubovisceral avulsions. All studies used the POP-Q system to stage POP. Except for Morgan et al. [28], who did not find a difference in anterior, apical, and posterior POP-Q data between women with and without major levator defects, all other ten studies did find a significant relation with women with pubovisceral avulsions reporting POP more often (Table 4). This relation was strongest with regard to the anterior [14, 17, 22, 24, 29, 31, 38] and central vaginal compartment [14, 17, 29, 31], but was less apparent for the posterior vaginal compartment [24, 29].

**Table 4:** POP anatomy and symptoms in women assessed for pubovisceral avulsions

First author	*n* with levator avulsion		Percentage of women of study population with levator avulsion	Conclusion
Abdool [29]	Bilateral	31	9 %	Cystocele and rectocele (on POP-Q) were associated with both unilateral and bilateral avulsion. Uterine prolapse was only related to bilateral avulsion
	Unilateral	45	12 %	
Adekanmi [23]	Cases:			Women with symptomatic POP ≥ stage II showed in 56 % of the cases a partial or complete avulsion. No avulsions were identified in nulliparous women
	Partial	18	26 %	
	Complete	21	30 %	
	Controls	0	0 %	
DeLancey [13]	Cases:			Major avulsions were statistically significant related to POP status (*p* < 0.001) and associated with an adjusted OR of 7.3 (95 % CI 3.9–13.6)
	Major	83	55 %	
	Minor	24	16 %	
	Controls:			
	Major	21	16 %	
	Minor	30	22 %	
Dietz [14]	Complete	181	23 %	Women with complete avulsions were twice as likely to have significant POP, especially cystocele and uterine prolapse
Dietz [38]	Complete	21	20 %	Women with a palpated avulsion showed more cystocele descent both on ultrasound and on POP-Q
Dietz [17]	Complete	46	14 %	Women with complete avulsions had higher grades of POP of the anterior and central compartment. There was no association between complete avulsion and POP symptoms
Dietz [31]	Complete	50	19 %	Defect score was associated with cystocele and uterine prolapse and POP symptoms
Dietz [33]	Complete	104	25 %	Women with a complete avulsion were more likely to have POP of the anterior compartment (*p* < 0.001)
Dietz [22]	Complete	226	30 %	A complete avulsion was strongly associated with symptoms of POP, significant POP on clinical assessment, and bladder descent on perineal ultrasonography (all *p* < 0.001)
Heilbrun [24]	Major	20	10 %	POP-Q points Ba and Bp were more often at or below the hymen in women with major avulsions compared to women with no or minor avulsions
Morgan [28]	Major	46	55 %	There was no difference in preoperative anterior, apical, and posterior POP-Q data between women with and without a major avulsion

*POP* pelvic organ prolapse, *n* number of patients, *POP-Q* Pelvic Organ Prolapse Quantification, *p value* *p* value for difference between avulsion and outcome measure assessed, *OR* odds ratio, *95 % CI* 95 % confidence interval, *Ba and Bp* most descended edge of anterior and posterior vaginal wall, respectively, relative to the hymen

Table 5 shows that recurrence of POP after surgery was related to pubovisceral avulsions in all available studies on the subject [28, 32, 34, 35, 37]. This association was merely seen in women with major pubovisceral avulsions as diagnosed by MR imaging or in women with a complete avulsion according to perineal ultrasonography. Wong et al. [37] have reported on the recurrence of POP in women with pubovisceral avulsions following anterior vaginal mesh surgery.

**Table 5:** Recurrence of POP after surgery in relation to pubovisceral avulsions

First author	*n* with levator avulsion		Percentage of women of study population with levator avulsion	Follow-up duration^a^	Conclusion
Dietz [32]	Complete	29	35 %	4.5 years [3–6.4]	Complete avulsion was associated with an RR of 3 to 4 for cystocele recurrence
Model [34]	Complete	156	21 %	NR	Complete avulsion was associated with an increased prevalence of significant POP and symptoms of POP after previous POP or anti- incontinence surgery
Morgan [28]	Major	46	55 %	42.3 days (12.0)	Women with major avulsions were less likely to have anterior compartment support at least 2 cm above the hymen after surgery compared to women with no or minor avulsions
Weemhoff [35]	Partial	59	39 %	31 months [14–50]	Fifty-two percent of women with anatomical recurrence of cystocele had a complete avulsion compared to 31 % of women without anatomical recurrence. There was no difference in anatomical recurrence in relation to partial avulsion
	Complete	63	41 %		
Wong^b^ [37]	Complete	83	38 %	2.1 years [6 weeks–5.6 years]	Complete avulsion was associated with an OR of 2.27 (95 % CI 1.23–4.21) for significant cystocele recurrence on ultrasound. This effect was significant for women after a specific type of mesh operation

*POP* pelvic organ prolapse, *n* number of patients, *SD* standard deviation, *NR* not reported, *RR* relative risk, *OR* odds ratio, *95 % CI* 95 % confidence interval

^a^Median [range] or mean (SD)

^b^Conference abstract, (so far) no full text article available

## Discussion

In this systematic literature review, we assessed the clinical relevance of diagnosing pubovisceral avulsions in women with pelvic floor dysfunction. Diagnostic methods of interest were MR imaging and perineal ultrasonography. The presence of pubovisceral avulsions was shown to be relevant with respect to POP symptoms and POP stage, especially in the anterior and central compartment. Recurrence of POP after surgery was also related to avulsions according to the available studies. There was no clear relation between pubovisceral avulsions and SUI, but in this respect, there might be a difference between minor and major defects [26]. Studies with regard to the relation between symptoms of anorectal dysfunction and pubovisceral avulsions yielded contradictory results.

Even though it has long been recognized that the levator ani muscle plays a critical role in pelvic organ support, in what way pregnancy and/or childbirth injure the pelvic floor has not been proven conclusively. Pelvic floor injury might be caused by compression, stretching or tearing of nerves, muscles, and/or connective tissue [42, 43]. Evaluation of the importance of muscle integrity has gone through an exponential growth over the past decade with the assessment of pubovisceral avulsions initially being performed using MR imaging. Therefore, this diagnostic method became the reference standard. The research group of Professor DeLancey was the first to standardize the evaluation of MR images [44]. However, nowadays a growing amount of studies use perineal ultrasonography to assess pubovisceral muscle integrity as this diagnostic method has the advantage over MR imaging of easier implementation in routine clinical care together with the benefits of significantly lower cost and superior availability. The standardization of perineal ultrasonography when evaluating pubovisceral avulsions was performed under the supervision of Professor Dietz [31]. Approximately half of the studies in this review focused on using MR imaging to detect pubovisceral avulsions while a slightly higher number of studies used perineal ultrasonography.

There was a notable variance in the naming for the subdivision of the levator ani muscle of interest for this review. In 2004, Kearney et al. [45] performed a literature search on the various descriptions and terminology for this muscle. They found that even though there was a great diversity regarding the terms chosen in the available literature, the number of origin and insertion pairs was relatively consistent among authors. Overall, the levator ani muscle comprises three subdivisions, namely, the iliococcygeal, puborectal, and pubovisceral muscles. The pubovisceral muscle includes the puboanal, puboperineal, and pubovaginal muscles and, together with the puborectal muscle, originates from the pubis [15]. Another frequently used term for the pubovisceral muscle is pubococcygeus muscle; however, this implies a connection between the pubis and coccyx while in fact the muscle originates from the pubis and inserts into the wall of the vagina and anorectum. It is thus our belief that pubovisceral muscle is the correct term to be used.

We had some difficulty to decide on the inclusion of one study by Adekanmi et al. [46]. The muscle injuries that were evaluated differed from injuries as described in all other studies with a distinction being made by Adekanmi et al. between central and lateral (endopelvic) fascial defects as well as changes in vaginal configuration after surgery. Based on subsequently published literature and correspondence with the authors, we came to the conclusion that the researchers had not studied pubovisceral avulsions as included in this review. The paper was therefore excluded.

The present review showed that the relation between POP and pubovisceral avulsions is eminent as all but one paper [28] found a significant difference in POP incidence (Table 4). This relation was strongest in the compartments with highest recurrence rates after POP surgery, namely, the anterior and central vaginal compartment. DeLancey et al. [13] were the only researchers to perform a case-control study with group matching for POP status in which, after multivariable regression, avulsion was still identified as an independent risk factor for POP. We were not able to further assess the correlation between avulsions and POP stage, POP surgeries, and POP recurrences from the accumulated data in this review. However, as up to 30 % of POP surgeries are currently performed due to relapse of POP [2], pubovisceral avulsions seem clinically relevant as an independent risk factor for POP. It has been suggested that women with pubovisceral avulsions might benefit from primary vaginal mesh surgery [47, 48]. However, Wong et al. [37] found that mesh implementation in these women did not fully compensate for the effect of pubovisceral avulsions on recurrence rates. Evaluating pubovisceral muscle integrity in appropriate clinical POP outcome studies will be needed to further lead the way, especially with regard to different strategies in surgical repair of various POP stages. Which diagnostic method, MR imaging or perineal ultrasonography, should be used remains to be assessed as well [49].

While the association between urinary symptoms and childbirth is beyond dispute [50], this is presumably not directly related to pubovisceral avulsions, since available studies have shown contradictory results. Women with pubovisceral avulsions were less likely to suffer from SUI (subjective and/or objective as confirmed by urodynamics) [33]. When pubovisceral avulsions were divided into major and minor avulsions, it was found that women with major defects are less likely and women with minor defects are more likely to have lower urinary tract symptoms [26]. The lack of a clear association between avulsions and SUI might be due to the fact that these avulsions do not seem to affect urethral mobility as much as they affect bladder support [51, 52]. Moreover, Morgan et al. [28] suggested that damage to the pudendal nerve could explain the difference in frequency of urinary symptoms between major and minor defects. They proposed that with a minor injury the preservation of one side of the muscle or parts of both sides can result in an asymmetry that alters reflexive responses of the urethra, bladder, and pelvic floor leading to symptom exacerbation, while a complete injury may be symmetrical leading to the absence of a motor and sensory reflex and therefore potentially having a less dramatic effect on symptoms. Electromyographic research to support this hypothesis is currently lacking.

The fact that pubovisceral avulsions are caused by vaginal delivery is beyond question and the same accounts for the occurrence of sphincter tears due to vaginal delivery. As the latter association is found to be related to symptoms of anorectal dysfunction, e.g., fecal incontinence (FI), together with the similar etiology of pubovisceral avulsions and sphincter tears, we expected a relation between symptoms of anorectal dysfunction and pubovisceral avulsions. This expectation is strengthened by the finding of Heilbrun et al. [24] that women with major levator defects have a higher prevalence of anal sphincter tears. Moreover, Lewicky-Gaupp et al. [25] found that older women with FI were more likely to have levator defects than women without FI (both younger and older) and that this association remained significant after correcting for external anal sphincter tears. Rodrigo et al. [36] reported a higher prevalence of RI among a group of women with pubovisceral avulsions. After multivariable analysis, this relation came on the account of hiatal area size on Valsalva and was not the effect of avulsions per se.

A limitation of this review was the heterogeneity of the available studies. Due to the variation in diagnostic methods used, study populations included, and outcome measures assessed, it was not possible to pool the available data into reliable relative risk factors for pubovisceral avulsions. To perform a patient-specific risk analysis and to ultimately individualize therapy for POP, it is our recommendation to evaluate the integrity of the pubovisceral muscle in those women who are most affected by the consequences of pubovisceral avulsions, i.e., women visiting tertiary urogynecological clinics. Therefore, both standardized questionnaires regarding symptoms of pelvic floor dysfunction and clinical examination, e.g., POP-Q, should be registered in this population.

Another limitation was that all studies but one [35] on perineal ultrasonography are coauthored by Professor Dietz (Sydney, Australia). With regard to the technique of MR imaging, Professor DeLancey (Ann Arbor, MI, USA) coauthored six of eight of the included studies. Both researchers are renowned experts within their field, but external validation therefore seems relevant and is awaiting. Little data are currently available on agreement between MR imaging and perineal ultrasonography in the evaluation of pubovisceral avulsions. Regarding this, Zhuang et al. [41] are the first to compare ultrasonography to MR imaging. They reported a substantial agreement for both agreement between methods (Cohen’s kappa = 0.79) and for agreement regarding the extent of the avulsion (Cohen’s kappa = 0.65). To further evaluate the agreement between MR imaging and perineal ultrasonography with regard to pubovisceral avulsions and to obtain the level of agreement between observers, the translabial 3D-ultrasonography for diagnosing levator defects (TRUDIL) study is currently being performed in the Netherlands [49].

Besides visualizing pubovisceral avulsions, these muscle defects can also be palpated. Using palpation, a pubovisceral avulsion is diagnosed if there is a detachment of the pubovisceral muscle from its insertion on the pubis [38]. Palpation can be easily incorporated in the standard gynecological examination, but it has been reported to have a considerable learning curve with only moderate agreement between different observers [38, 40]. No studies could be identified that used palpation of the pubovisceral muscle defect as a diagnostic method solely. Three studies have, however, been published on the agreement between palpation and ultrasonography or MR imaging [38–40]. In 2006, Dietz et al. [39] evaluated palpation versus perineal ultrasonography in a cohort of 54 patients and found poor agreement (Cohen’s kappa = 0.098) between both methods with only two avulsions diagnosed by both methods. The agreement between two observers performing palpation of the muscle defect and an independent blinded reviewer of ultrasonographic data showed moderate and fair agreement, respectively [38]. Agreement between palpation and MR imaging was reported to be moderate (Cohen’s kappa = 0.444) [40]. In the latter study, a pubovisceral avulsion was detected by both methods in only 3 of 24 women. We believe that this might be explained by the fact that palpation appears to rely more on the comparison of findings with a supposedly intact contralateral side hereby making bilateral defects much more difficult to detect digitally than on imaging. Overall, it seems that even though palpation of the muscle defect appears to be the easiest diagnostic method to implement in routine clinical care, the value of this method is limited. Implementation of either perineal ultrasonography or MR imaging in the diagnostic workup of women with complaints of pelvic floor dysfunction appears more feasible and of added value.

## Conclusion

In conclusion, a clear relation exists between visualized pubovisceral avulsions and POP stage and symptoms of POP. Recurrence rates after POP surgery were also reported to be higher among women with this prevalent anatomical defect. The association between pubovisceral avulsions and urinary symptoms, and symptoms of anorectal dysfunction, was less apparent.

## Appendix 1: full PubMed literature search terms

((((((((((magnetic resonance imaging)) OR ("MR imaging")) OR ("MR studies")) OR ("MR study")) OR (MRI))) OR ((((((((ultrasonography)) OR (ultrasonograph*)) OR (ultrasonic imaging)) OR (echograph*)) OR (sonograph*)) OR (ultrasound)) OR (echo))) OR (((((physical examination)) OR (palpation)) OR (digital detection)) OR (medical examination)))) AND (((((((pelvic floor)) OR (levator ani)) OR (pubovisc*)) OR (pubococ*)) OR (puborect*)) OR (puboperine*))

## Appendix 2: full Embase literature search terms

((exp nuclear magnetic resonance imaging/ or (magnetic and resonance and imaging) or (MR stud*) or MRI) OR (exp echography/ or ultrasonic imaging or ultrasound or ultrasonograph* or echograph* or sonograph* or echo) OR (exp physical examination/ or exp medical examination/ or digital detection or palpation or physical examination or medical examination)) AND (exp levator ani muscle/ or levator ani or pelvic floor or pelvis floor or pubovisc* or puborect* or pubococ* or puboperine*)

## Appendix 3: full CINAHL literature search terms

((MH "Magnetic Resonance Imaging" or (magnetic and resonance and imaging) or MRI or MR Imaging or MR studies or MR study or MR stud*) or (MH "Ultrasonography + " or ultrasonography or ultrasound or echo or ultrasonic imaging or ultrasonograph* or sonograph* or echograph*) or (MH "Physical Examination + " or physical examination or medical examination or palpation or digital detection)) and (MH "Pelvic Floor Muscles" or levator ani or pelvic floor or pelvis floor or pubovisc* or pubococ* or puborect*)

## Abbreviations

POP: Pelvic organ prolapse
UI: Urinary incontinence
MR: Magnetic resonance
3-D: Three-dimensional
4-D: Four-dimensional (3-D + temporal information)
POP-Q: Pelvic Organ Prolapse Quantification
TUI: Tomographic ultrasound imaging
SUI: Stress urinary incontinence
RI: Rectal intussusception
FI: Fecal incontinence
TRUDIL: Translabial 3D-ultrasonography for diagnosing levator defects
